# Behavioral and Morphological Adaptations of Tortoise Tick *Hyalomma aegyptium* to *Testudo graeca*: Evidence for Complex Evolutionary History

**DOI:** 10.1002/ece3.71995

**Published:** 2025-08-14

**Authors:** Sirri Kar, Baris Donmez, Bugrahan Regaip Kilinc, Zafer Sakaci, Sengul Talay, Dennis Bente, Agustin Estrada‐Peña

**Affiliations:** ^1^ Department of Biology Tekirdag Namik Kemal University Tekirdag Turkey; ^2^ Department of Microbiology and Immunology, Galveston National Laboratory University of Texas Medical Branch Galveston Texas USA; ^3^ Department of Animal Health, Faculty of Veterinary Medicine University of Zaragoza Zaragoza Spain

**Keywords:** coevolution, intraspecific interaction, parasite–host adaptation, population dynamic, tick, tortoise

## Abstract

In vector arthropods, the host relationship plays a central role in population dynamics and is crucial for determining the current and future course of vector ecology and the eco‐epidemiology of vector‐borne diseases, particularly under the influence of climate and environmental changes. However, since this relationship is driven by complex ecological cascades, accurately characterizing its attributes, particularly in a cause‐and‐effect context, remains challenging, leaving substantial gaps in understanding. In this study, we examined the infestation characteristics of the tick species *Hyalomma aegyptium* on its specific host, the spur‐thighed tortoise *Testudo graeca* , to investigate the principles of behavioral and morphological adaptation and its ecological consequences. A field study was conducted in 2021 and 2022 in Turkish Thrace to obtain phenology‐based data under natural conditions. During the survey, a total of 20,933 ticks were examined on 878 tortoises, with 96.1% prevalence, 24.8 ± 30.6 intensity, and 23.8 abundance. The analyses revealed that all infestation traits were directly influenced by the tick's developmental stage, sex, and morphological characteristics, as well as the tortoise's sex, size, behavioral traits, and phenological patterns. The comprehensive evaluation of behavioral and morphological traits demonstrated that several features and behaviors in *H. aegyptium* are highly specialized to permit *T. graeca* infestation. All these traits seem evolutionarily driven to shield the tick from environmental and feeding‐related challenges while minimizing the infestation's life‐threatening pressure on the host. Although these remarkable adaptation characteristics suggest a deep‐rooted coevolutionary background, some critical discrepancies in the fundamentals of host–parasite interactions make it more plausible that the primary speciation process of *H. aegyptium* had already occurred in an extinct giant tortoise species before its adaptation to the genus *Testudo*.

## Graphical abstract

In this study, we examined the infestation characteristics of the tick species *Hyalomma aegyptium* on its specific host, the spur‐thighed tortoise *Testudo graeca* , to investigate the principles of behavioral and morphological adaptation and its ecological consequences. The comprehensive evaluation of behavioral and morphological traits demonstrated that several features and behaviors in *H. aegyptium* are highly specialized to permit *T. graeca* infestation. All these traits seem evolutionarily driven to shield the tick from environmental and feeding‐related challenges while minimizing the infestation's life‐threatening pressure on the host. Although these remarkable adaptation characteristics suggest a deep‐rooted coevolutionary background, some critical discrepancies in the fundamentals of host–parasite interactions make it more plausible that the primary speciation process of *H. aegyptium* had already occurred in an extinct giant tortoise species before its adaptation to the genus Testudo.

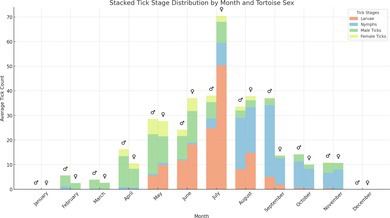

## 1 Introduction

Parasite–host relationship in ticks is one of the most important components of population dynamics and is a crucial determinant of the present and future status of populations and tick‐borne disease eco‐epidemiology in an environmentally dynamic world (McCoy et al. 2013; Dietrich et al. 2014; Kar and Keles 2021). However, since the parasite–host system in ticks is shaped by multiple interacting factors—related to the tick, host, and extrinsic environmental conditions such as climate and abiotic biogeographic factors (Kempf et al. 2009; Petney et al. 2012; Cangi et al. 2013) it is particularly challenging to accurately determine its properties and ecological significance, especially in a cause‐and‐consequence context.

Parasitism is one of the most complex forms of interspecies interactions and is often associated with ancient coevolution and even cospeciation (Papkou et al. 2016; Ebert and Fields 2020). The close interaction between parasites and hosts is thought to have triggered adaptive radiation, making parasites among the most diverse organisms on Earth (Bush et al. 2019). Although this phenomenon has been well documented in several parasitic systems (Schmid‐Hempel 2021), there is still no clear consensus regarding the origins, strength, and ecological consequences of host specialization in ticks. However, from the few examples studied, it seems that ticks and hosts coevolved but did not cospeciate (Estrada‐Peña et al. 2016, 2021). According to one mainstream perspective, host specificity is an intrinsic trait in ticks, with the tick‐host relationship being deeply rooted in coevolution and playing a pivotal role in tick speciation (Hoogstraal and Aeschlimann 1982; Hoogstraal and Kim 1985). Conversely, another dominant view suggests that environmental factors are the primary drivers of parasitism, long‐term coevolution is not always necessary, and most ticks function as host generalists (Balashov 2004; Cumming 2002; Klompen et al. 1996; Cuervo et al. 2021). The observation that rapid adaptive microevolution can trigger rapid adaptive species radiation in parasites (Bush et al. 2019) supports the argument that a long‐term evolutionary history is not always mandatory for the development of existing tick‐host relationships. However, this does not exclude the possibility that a well‐established coevolutionary process occurring at some point in history may have contributed to the acquisition of species‐specific characteristics. The fidelity of host association in ticks is, of course, debatable, as different life stages of the same species may feed on other hosts, some stages may parasitize various hosts within the same region, and certain ticks can adapt to phylogenetically distant hosts across different geographic areas (Klompen et al. 1996; Cumming 2002). However, most tick species deviate from at least one of these general patterns (Estrada‐Peña et al. 2017). Moreover, the ability to utilize alternative hosts based on environmental conditions may result from genetically preserved adaptability rather than a lack of host specificity.

Arthropods exhibit remarkable plasticity and rapid evolutionary responses, a trait that can be advantageous for survival in changing environments. However, because adaptation to conditions in one period may increase sensitivity to conditions in the next period, this adaptability is not universally beneficial, particularly in unpredictably fluctuating conditions (Chevin et al. 2010; Oostra et al. 2018; Bonamour et al. 2019). Therefore, arthropods tend to maintain species‐specific fundamental genomic structures, and many species benefit from simple genetic mechanisms, such as reversible mutations and genetic drift (Charlesworth and Jain 2014), particularly in the early stages of environmental change, and they avoid excessive genomic modifications to prevent deleterious genetic perturbations (Moczek et al. 2011). In ticks, the repetitive genomic structure may provide a means for such genetic flexibility, allowing for rapid adaptation to environmental changes and new hosts (McCoy et al. 2013). However, the specific pathways through which these mechanisms operate over extended adaptation periods likely vary between species and environmental conditions.

The tortoise tick *Hyalomma aegyptium* (Linnaeus, 1758), which is naturally distributed from Morocco in northwestern Africa to Kyrgyzstan in Central Asia (Rubel 2025), is a confirmed and/or suspected vector for *Coxiella burnetii* (the causative agent of Q fever) (Široký et al. 2010; Khademi et al. 2023), *Hemolivia mauritanica* (a blood parasite of reptiles) (Paperna 2006), *Hepatozoon kisrae* (a blood parasite of reptiles) (Paperna et al. 2002), Crimean‐Congo hemorrhagic fever virus (Kar et al. 2020), Tamdy virus, an unclassified nairovirus (Ergunay et al. 2020), *Rickettsia aeschlimannii* , *Rickettsia africae* (Gargili et al. 2012), *Borrelia burgdorferi* sensu lato (Kar et al. 2013), *Anaplasma phagocytophilum* , *Ehrlichia canis* (Pastiu et al. 2012), *Babesia microti* (Mumcuoglu et al. 2022), and *Francisella tularensis* (Tukmechi et al. 2023). This three‐host tick exhibits a high degree of host specificity, with all three stages (larva, nymph, and adults) predominantly feeding on tortoises belonging to the genus *Testudo* (Rubel 2025), such as *T. graeca* , *T. hermanni* , *T. marginata* (Siroký et al. 2006), and *T. horsfieldii* (Kaiser and Hoogstraal 1963). Immature stages have been occasionally found on humans and other animals such as birds, hedgehogs, and hares (Hoogstraal and Kaiser 1960; Gargili et al. 2010; Kar et al. 2017). Due to its strong host specificity, the distribution of this species is largely confined to the Mediterranean Basin, North Africa, and Southwest Asia—closely mirroring the distribution of its host (Hoogstraal and Kaiser 1960). The genus *Hyalomma* Koch, 1844 consists of 27 recognized species spanning the Palearctic, Indo‐Malaysian, and Afrotropical regions (Estrada‐Peña et al. 2017). *Hyalomma* species are large‐bodied ticks with high morphological and genetic similarity (Estrada‐Peña et al. 2017; Sands et al. 2017; Hosseini‐Chegeni et al. 2019). Despite these similarities, species within the genus exhibit significant ecological differences, including variations in host range, habitat preference, and seasonal activity patterns (Estrada‐Peña et al. 2017; Akyildiz et al. 2021; Kar et al. 2021). Furthermore, the evolutionary history and speciation processes of *Hyalomma* remain mostly enigmatic (Hoogstraal and Aeschlimann 1982; Sands et al. 2017; Estrada‐Peña and de la Fuente 2018; Chitimia‐Dobler et al. 2023). The host association of *H. aegyptium* poses an evolutionary paradox. While most *Hyalomma* species parasitize large‐bodied mammals such as cattle, horses, and camels, *H. aegyptium* has adapted to small, slow‐moving, narrow‐ranging, poikilothermic hosts of the *Testudo* genus. *Hyalomma* ticks are fast‐moving, highly mobile, and capable of significant blood uptake; their adaptation to a small, ecologically restricted host likely involves specialized mechanisms that ensure a balanced ecological relationship between parasite and host.

This study aimed to examine the long‐term phenological patterns of the *H. aegyptium* ‐ *T. graeca* relationship under natural field conditions in Turkish Thrace, a highly endemic region for both species. Specifically, we sought to (i) identify morphological, physiological, and behavioral parameters influencing infestation dynamics and (ii) assess the impact of intraspecific interactions and density‐dependent regulation on infestation patterns and population dynamics. The results are evaluated and further discussed in terms of possible coevolutionary background.

## 2 Materials and Methods

### 2.1 Study Area

The study was conducted in Turkish Thrace (41°58′ N, 27°22′ E at the central point), the European part of Turkey, which has high *Testudo graeca* and *Hyalomma aegyptium* population densities (Kar et al. 2020). The fieldwork was carried out in 10 foci distributed over an area of approximately 2000 km^2^, mainly located in the Marmara Sea basin, where *T. graeca* population density is high.

### 2.2 Study Approach and Sampling Method

The field study was carried out monthly from January 2021 to May 2022. In order to examine a sufficient number of tortoises for each month, at least three foci were visited monthly. To avoid re‐examining the same tortoise within a single month, during a single survey at a focus, each site was visited only once per month, and tortoises were marked immediately after examination using a temporary marker.

Tortoises were located while walking at the foci and were captured by hand. Each tortoise was sexed based on morphological criteria (Fritz et al. 2007). The carapace length was measured, and individuals smaller than 10 cm were classified as juveniles, as their secondary sexual characteristics were not fully developed. The body of each tortoise was examined across four anatomical regions: cranial (anterior body parts, including legs, head, neck, shoulders, and other exposed skin areas), caudal (posterior body parts, including hind legs, tail, and surrounding skin), carapace (dorsal shell), and plastron (ventral shell).

Ticks attached to different body parts were counted and recorded separately for each tortoise, including larvae, nymphs, males, and females. To prevent spatiotemporal alterations in natural tick population dynamics, ticks were not collected, and the tortoises were released at the exact location where they were found; however, subsamples of ticks from different developmental stages were collected for morphological species identification (Hoogstraal 1956; D. A. Apanaskevich 2003), prioritizing macroscopically distinct individuals. The subsamples taken for this purpose were brought to the laboratory alive in tubes and were directly subjected to morphological examination.

### 2.3 Statistical Analysis

Infestation metrics prevalence (percentage of infested tortoises), intensity (mean tick count per infested individual), and abundance (mean tick count per examined individual) were computed with respect to host size, sex, and seasonal variation. Differences in tick prevalence across sex categories were assessed using the chi‐square test of independence. Tick intensity was compared between sexes using the Mann–Whitney *U* test due to non‐normal distributions. Two‐Way ANOVA was employed to examine the effects of month, sex, and their interaction on tick intensity, followed by Tukey HSD for post hoc comparisons. Pearson correlation was used to assess the association between carapace length and tick burden across developmental stages. Time series analyses explored seasonal patterns in infestation. Independent *t*‐tests and additional Two‐Way ANOVAs were conducted for stratified comparisons of tick stages by host sex and size. To investigate attachment site preferences and mating behaviors, correlation analyses were performed between tick developmental stages and anatomical regions, as well as between developmental stages and mating female counts. Heatmaps were generated for visualization. All statistical analyses were conducted using Python (version 3.11, released in 2022).

## 3 Results

### 3.1 Tortoises

A total of 878 tortoises were examined for ticks, with the following sex distribution: 287 females (32.7%), 568 males (64.7%), and 23 juveniles (2.6%). The mean carapace length (±SD, range) in cm was 17.4 ± 2.8 (10–23) for females, 15.8 ± 1.9 (10–21) for males, and 8.3 ± 1.5 (5–9.5) for juveniles. The overall mean carapace length across all individuals was 16.1 ± 2.7 cm (5–23 cm). The female‐to‐male ratio was 0.51:1 (287/568), and a male‐biased sex distribution was consistently observed across all months.

Feeding and mating behaviors were not observed during early spring. However, activity levels increased in the following months, peaking during the reproductive season, and ceased by November.

The analyses showed that the number of male and female tortoises encountered in the field was found to differ significantly across months, with a notable increase in the proportion of males during April and May (*χ* ^2^ = 46.29, *p* = 0.00027) (Figure S1). This pattern suggests potential associations with seasonal behavior. Analyses of tortoise size revealed significant differences in straight carapace length across months (*F* = 2.78, *p* = 0.0033; *H* = 21.75, *p* = 0.0097) (Figure S2). A wider range of sizes was observed in the spring months, particularly in April and May, indicating the presence of both juvenile and adult individuals. However, Tukey HSD post hoc analysis did not identify specific significant differences between individual months. Differences in activity levels may influence these variations in size distributions. Categorical analyses demonstrated that adult tortoises dominate the total population, with the most notable monthly fluctuations observed within this group. The counts for the juvenile group remained minimal, preventing further meaningful conclusions for this group.

### 3.2 General Pattern of Infestation

In 878 examined tortoises, a total of 20,933 ticks were recorded, all of which were identified as *H. aegyptium* . The number of larvae, nymphs, females, and males was 6523 (31.2%), 4941 (23.6%), 2430 (11.6%), and 7039 (33.6%), respectively. The prevalence, mean abundance, and intensity (±SD) of total ticks were 96.1%, 23.8, and 24.8 ± 30.6, respectively. The minimum intensity was one, while the maximum number of ticks on a tortoise was recorded in June with 263 ticks (210 larvae, 25 males, and 28 females) (Tables 1 and 2).

**TABLE 1:** Monthly infestation characteristics of *Hyalomma aegyptium* in tortoises.

Month	No. tortoises examined				Infestation characteristics of total ticks^a^		
	Female	Male	Juvenil	Total	Prevalence (%)	Intensity ±SD (range)	Abundance
January	Tortoises not found						
February	2	9	—	11	90.9	5.0 ± 5.0 (1–18)	4.6
March	7	47	—	54	77.8	3.8 ± 3.0 (1–13)	2.9
April	51	103	2	156	94.9	14.4 ± 10.6 (1–53)	13.7
May	95	138	14	247	95.1	27.2 ± 24.8 (2–213)	25.9
June	35	46	—	81	100	29.7 ± 44.0 (1–263)	29.7
July	24	53	3	80	100	46.8 ± 54.8 (1–245)	46.8
August	28	43	2	73	100	34.7 ± 29.4 (3–173)	34.7
September	22	53	—	75	100	30.3 ± 25.5 (1–133)	30.3
October	20	54	2	76	98.7	12.9 ± 13.6 (1–95)	12.7
November	3	22	—	25	100	10.9 ± 7.9 (1–36)	10.9
December	Tortoise not found						
Total	287	568	23	878	96.1	24.8 ± 30.6 (1–263)	23.8

^a^ Prevalence: ratio of infested tortoises to all tortoises examined; intensity: amount of ticks/number of tortoises infested; abundance: number of ticks/total number of tortoises examined.

**TABLE 2:** Monthly abundance of *Hyalomma aegyptium* larvae (L), nymphs (N), males (M), and females (F) in tortoises.

Month	Abundance^a^											
	All tortoises				Male tortoises				Female tortoises			
	L	N	M	F	L	N	M	F	L	N	M	F
January	Tortoise not found											
February	—	0.9	3.6	—	—	1.1	3.9	—	—	—	2.5	—
March	—	0.1	2.8	0.04	—	0.1	2.8	0.04	—	—	2.9	—
April	0.01	0.3	10.4	2.4	0.01	0.9	12.3	2.7	—	0.6	6.9	2.0
May	6.8	0.4	12.7	5.6	5.4	0.7	15.6	6.0	9.4	0.9	10.3	5.9
June	14.6	0.6	10.9	3.7	11.8	0.5	9.4	2.6	18.2	0.7	12.8	5.3
July	31.9	5.5	6.9	2.5	24.8	4	6.6	2.7	50.4	9.1	8.5	2.5
August	10.4	19.7	2.9	1.7	8.1	21	3	1.6	14.8	18.4	3	1.7
September	4.1	23.8	2.2	0.3	5.1	29.2	2.6	0.3	1.8	10.8	1.1	0.05
October	0.4	9.8	2.5	0.07	0.4	10.6	2.9	0.1	0.2	8.1	1.7	0.05
November	0.3	6.5	3.8	0.2	0.4	6.3	4	0.3	—	8	2.7	—
December	Tortoise not found											
Total	7.4	5.6	8.0	2.8	5.7	6.3	8.6	2.6	11.2	4.5	7.5	3.3

^a^ Abundance: total number of ticks counted/total number of tortoises examined.

For larvae and nymphs, the highest prevalence was 76.3% (July) and 98.7% (September), while the lowest prevalence was 0.6% (April) and 7.1% (March), respectively. For larvae, the highest intensity was 41.8 ± 53.0 (range: 1–210) in July, while the lowest was 1.0 in April. For nymphs, the highest intensity was 24.1 ± 21.8 (range: 1–128) in September, while the lowest was 1.5 ± 1.0 (range: 1–3) in March (Table S1).

**FIGURE 1:**
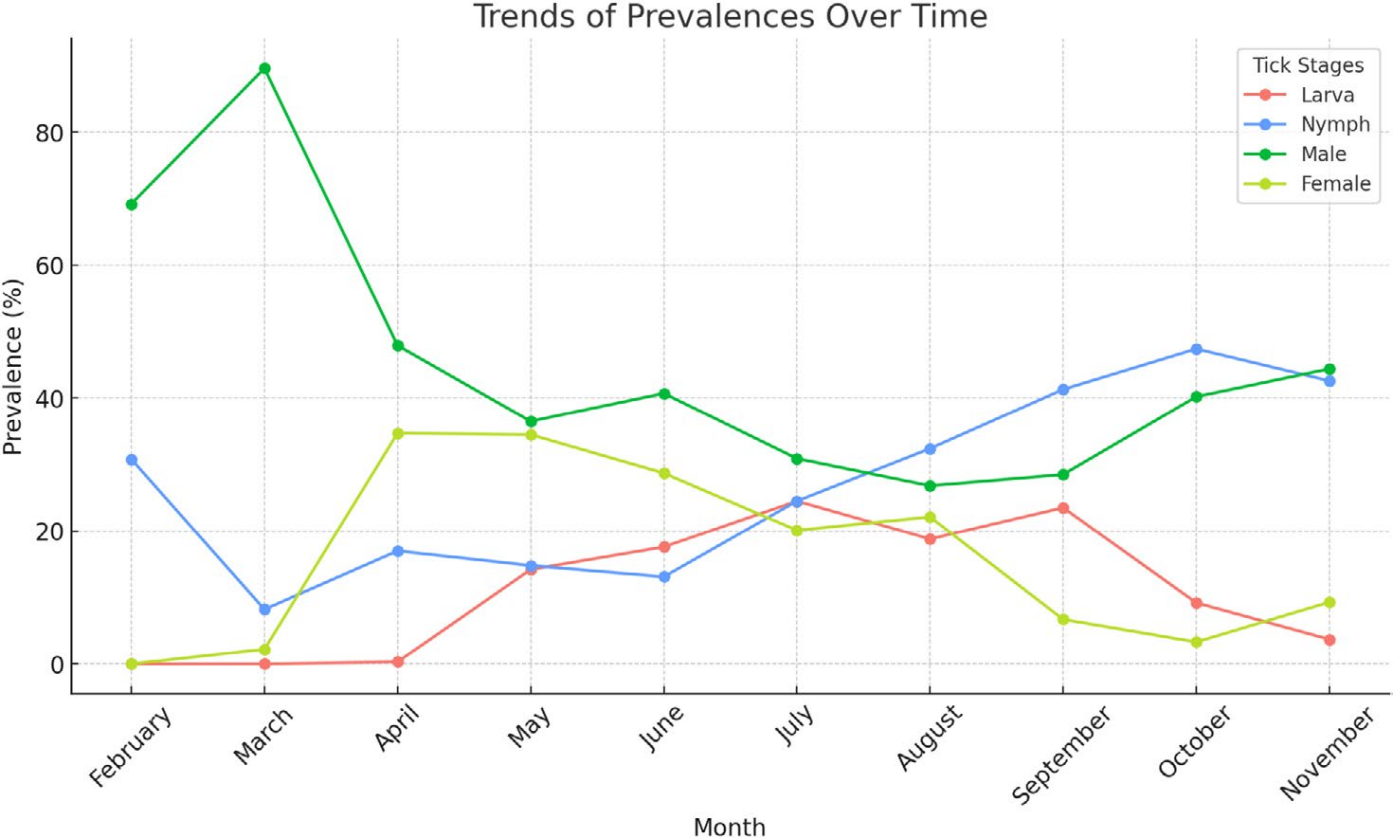
Monthly prevalence of tick development stages in tortoises.

**FIGURE 2:**
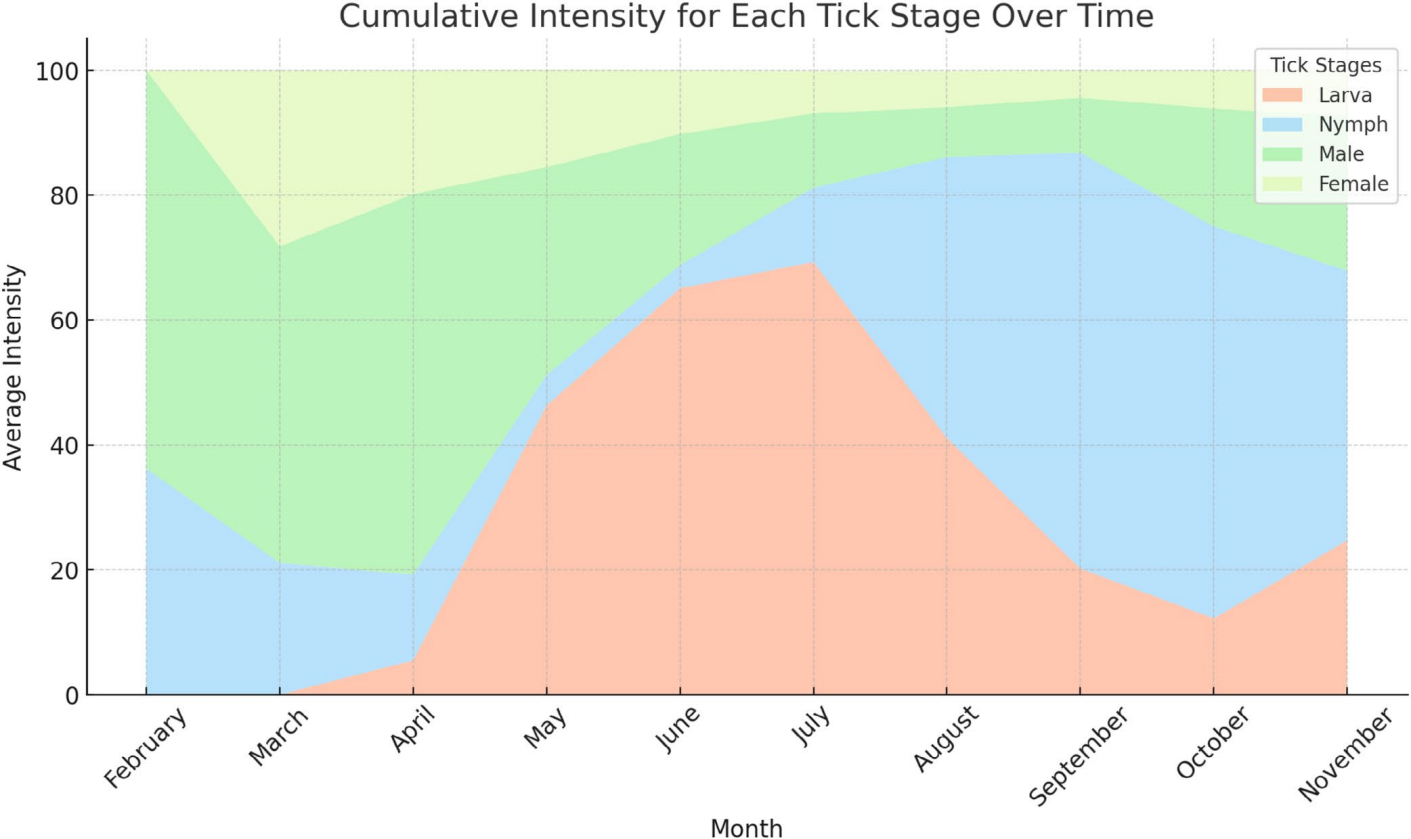
Monthly course of cumulative intensity for tick development stages.

The prevalence, abundance, and intensity of adult ticks were 89.5%, 10.8, and 12.1 ± 12.0 (range: 1–86), respectively. Overall prevalence and intensity were 87.4% and 9.2 ± 8.6 (range: 1–70) for male ticks and 55.8% and 5.0 ± 4.8 (range: 1–30) for female ticks. Male ticks were observed in all months, with the highest prevalence recorded in June (100%) and the highest intensity recorded in May (14.3 ± 10.2, range: 1–70). The lowest prevalence was 68.0% (September) and the intensity was 3.1 ± 3.4 (range: 1–24) (October). For female ticks, the highest prevalence and intensity were recorded in May (83.8%) and 6.7 ± 5.4 (range: 1–30), while the lowest abundance was 1.9% (March) and the lowest intensity was 1.0 ± 0.0 (range: 1–1) (October) (Table S2) (Figures 1 and 2).

### 3.3 Correlation Between Tick Infestation and Sex of the Tortoises

In 23 juvenile tortoises (indeterminate sex and less than 10 cm), which were encountered over the months of April, May, July, August, and October, 74 larvae, 40 nymphs, 10 males, and 13 females were found; mean intensity (±SD and range of tick number) of these stages was 6.2 ± 5.4 (1–20), 5.0 ± 5.3 (1–18), 1.7 ± 1.5 (1–5), and 4.3 ± 0.9 (3–5), respectively. Male tortoises have a higher prevalence and intensity of nymphs, male, and total ticks; however, larvae and female ticks showed higher indexes in female tortoises (Tables S3 and S4). Tick prevalence significantly differed between sexes, with male tortoises exhibiting a higher prevalence compared to females and juveniles (*χ* ^2^ = 20.48, *p* < 0.001). Tick intensity also showed notable differences. Female tick counts were significantly higher on female tortoises than on males (*p* = 0.018), suggesting a sex‐specific preference for female tortoises. In addition, intensity was higher in the female tortoises (Table 3). In male tortoises, overall prevalence and intensity (mean ± SD and range of tick number) of larvae, nymphs, male, female, and total adult ticks were 29.9 and 19.1 ± 27.6 (1–210), 55.8 and 11.4 ± 16.4 (1–128), 90.1 and 9.5 ± 9.0 (1–70), 54.2 and 4.7 ± 4.4 (1–27), and 91.9 and 12.2 ± 12.1 (1–86). These values in female tortoises were 34.2 and 32.6 ± 49.7 (1–210), 51.9 and 8.7 ± 15.4 (1–110), 86.8 and 8.6 ± 7.7 (1–39), 62.4 and 5.3 ± 5.4 (1–30), and 89.2 and 12.1 ± 11.7 (1–69), respectively.

**TABLE 3:** Infestation characteristics of different tick stages in male and female tortoises.

Ticks	Prevalence^a^		Abundance^a^		Intensity^a^	
	Male tortoises	Female tortoises	Male tortoises	Female tortoises	Male tortoises	Female tortoises
Larva	29.9	34.2	5.7	11.1	19.1 ± 27.6	32.6 ± 49.7
Nymph	55.8	51.9	6.3	4.5	11.4 ± 16.4	8.7 ± 15.4
Male	90.1	86.8	8.6	7.5	9.5 ± 9.0	8.6 ± 7.7
Female	54.2	62.4	2.6	3.3	4.7 ± 4.4	5.3 ± 5.4
Total adults	91.9	89.2	11.2	10.8	12.2 ± 12.2	12.1 ± 11.7
Total ticks	96.8	96.2	23.2	26.5	24.0 ± 25.3	27.5 ± 39.6

^a^ Prevalence: ratio of infested tortoises to all tortoises examined; intensity: amount of ticks/number of tortoises infested; abundance: number of ticks/total number of tortoises examined.

**FIGURE 3:**
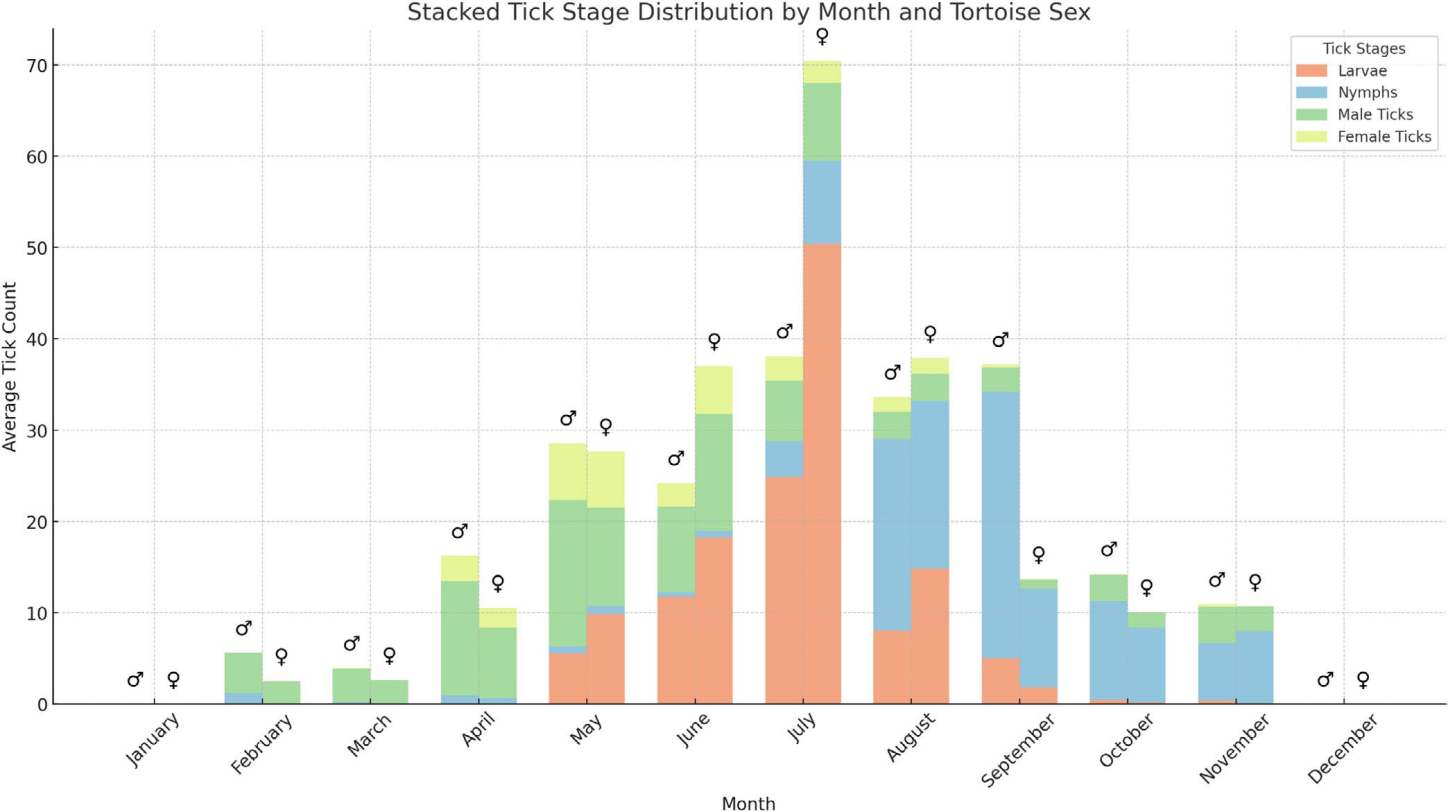
Stacked tick stage distribution by month and tortoise sex.

Seasonal variations in tick burden were evident, with significant main effects of month (*p* < 0.05) and tortoise sex (*p* < 0.05) on tick intensity; this seasonal trend was confirmed by time series analysis. However, the phenological pattern of the host sex‐dependent infestation characteristic exhibited an irregular monthly course. Seasonal variations indicated that female ticks preferred male tortoises in spring and autumn, while a significant aggregation of female tortoises was observed in the summer months, particularly in May and June (Figure 3).

### 3.4 Correlation Between Tick Infestation and Size of the Tortoises

**FIGURE 4:**
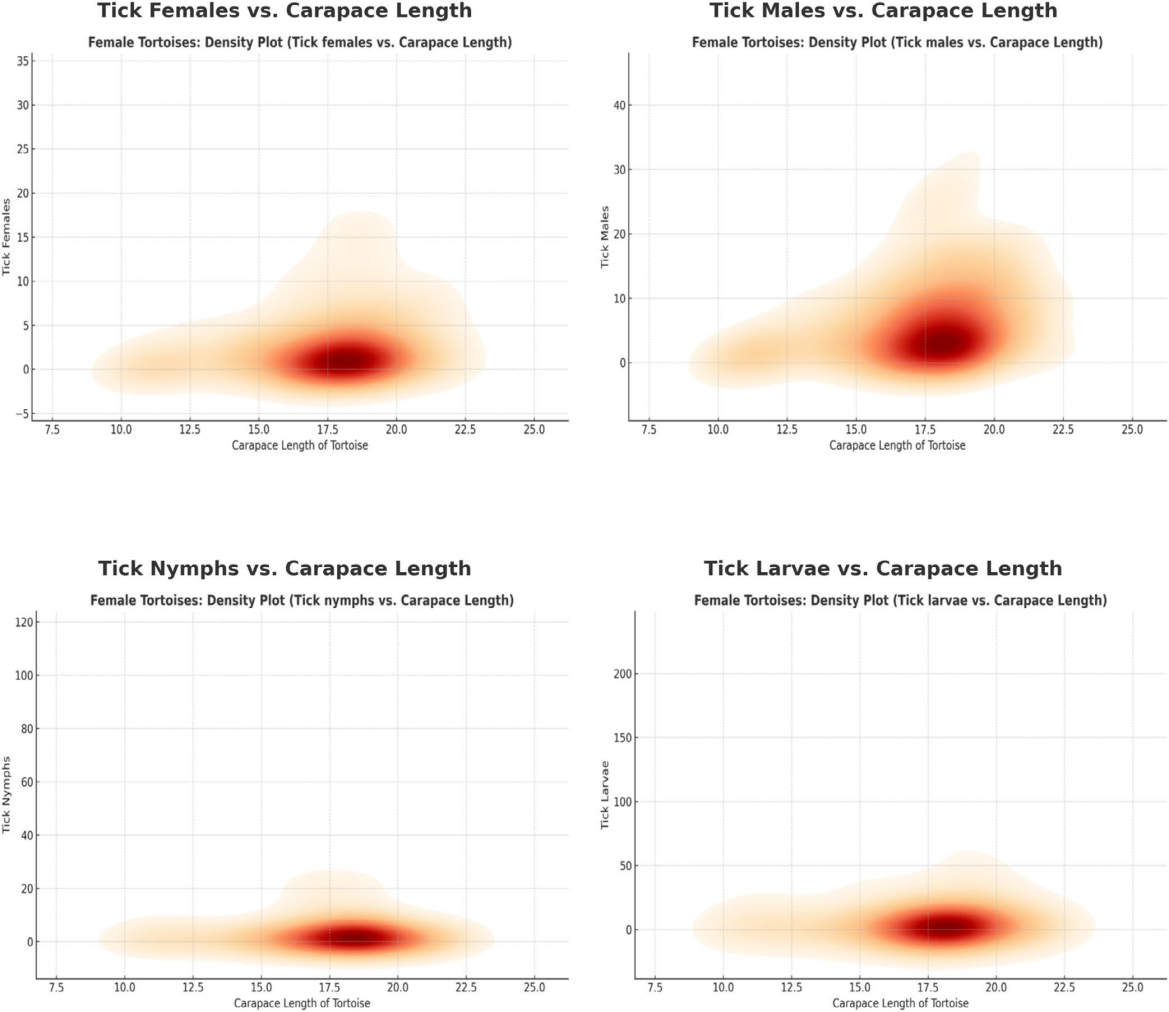
Comparison of tick distribution in female tortoises based on carapace length.

**FIGURE 5:**
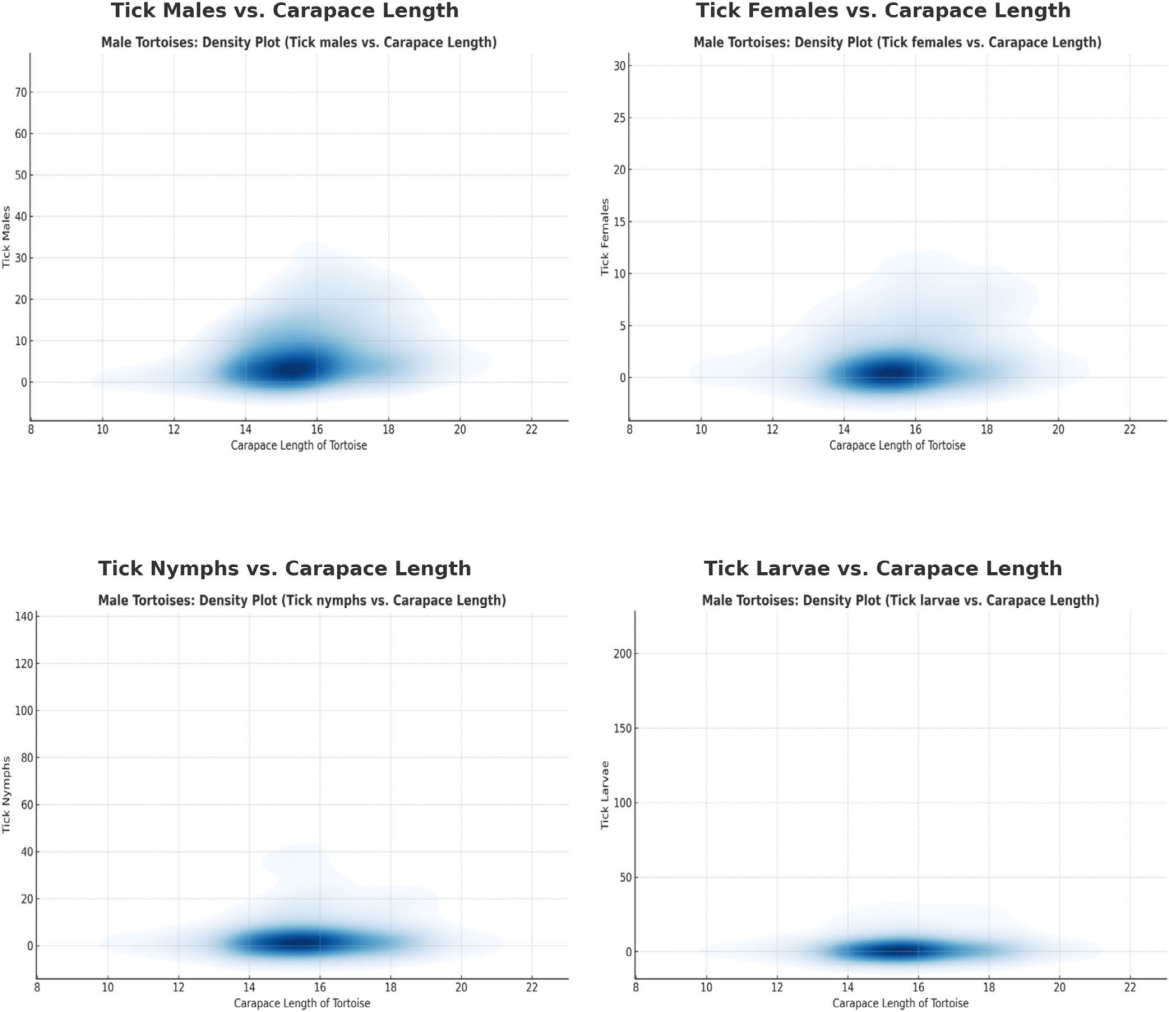
Comparison of tick distribution in male tortoises based on carapace length.

The correlation analysis between tortoise size (carapace length) and tick infestation revealed significant yet variable relationships across tick developmental stages (Figure S3). Pearson correlation coefficients indicated a weakly positive correlation between tortoise size and tick infestation intensity, particularly for adult ticks (*r* = 0.2641, *p* < 0.0001). Both male (*r* = 0.2342, *p* < 0.0001) and female tortoises (*r* = 0.2798, *p* < 0.0001) exhibited a similar trend, where larger tortoises harbored higher numbers of adult ticks (Figures 4 and 5). In contrast, the correlation between tortoise size and larval counts (*r* = 0.1236, *p* = 0.0002) and nymph counts (*r* = 0.0729, *p* = 0.0308) was considerably weaker. These findings suggest that immature tick stages are less influenced by host size, although tortoises with carapace lengths between 14 and 20 cm appear to be more frequently targeted by adult ticks, as evidenced by the concentration of tick counts within this size range.

### 3.5 Attachment Site Segregation Patterns of the Ticks

Of the total 6523 larvae, 6275 (96.2%) were found on the skin of the anterior cavities of the tortoises, while 248 (3.8%) were located in the posterior cavities. Among the 4941 nymphs, 4056 (82.1%) were observed in the anterior region, whereas 885 (17.9%) were in the posterior region. The number of the male ticks at the anterior, posterior, carapace, and plastron were 1069 (15.1%), 5904 (83.9%), 47 (0.7%), and 19 (0.3%); and the number of the females on the same sites were 459 (18.9%), 1920 (79.0%), 35 (1.4%), and 16 (0.7%), respectively.

In the anterior region, adult ticks were primarily found on the skin between the extremities and the shell, with rare occurrences on the dorsal part of the head, neck, legs, and pods (only two females). In the posterior region, adult ticks were also mostly located on the skin between the extremities and the shell, with occasional occurrences on the legs and tail. Male ticks were rarely observed on the extremities and, unlike females, tended to aggregate in specific areas, particularly at the skin‐shell junction of the caudal axillar depression, a grooved region facilitating leg movement (Figure S4).

The 73.4% of adult ticks on the carapace were at the suture line between the last vertebral (= centrale) scute and postcentral (= supracaudal) scute; the 17.5% were collected at the suture line between the last pleural (= costal) and marginal scute, and five female ticks were at shell scars on dorsal parts of the carapace in two tortoises. On the plastron, one male and two female ticks were at a depression at the central suture line level between the pectoral and abdominal scute, and all the other ticks were at the suture line between the abdominal and femoral scute.

**FIGURE 6:**
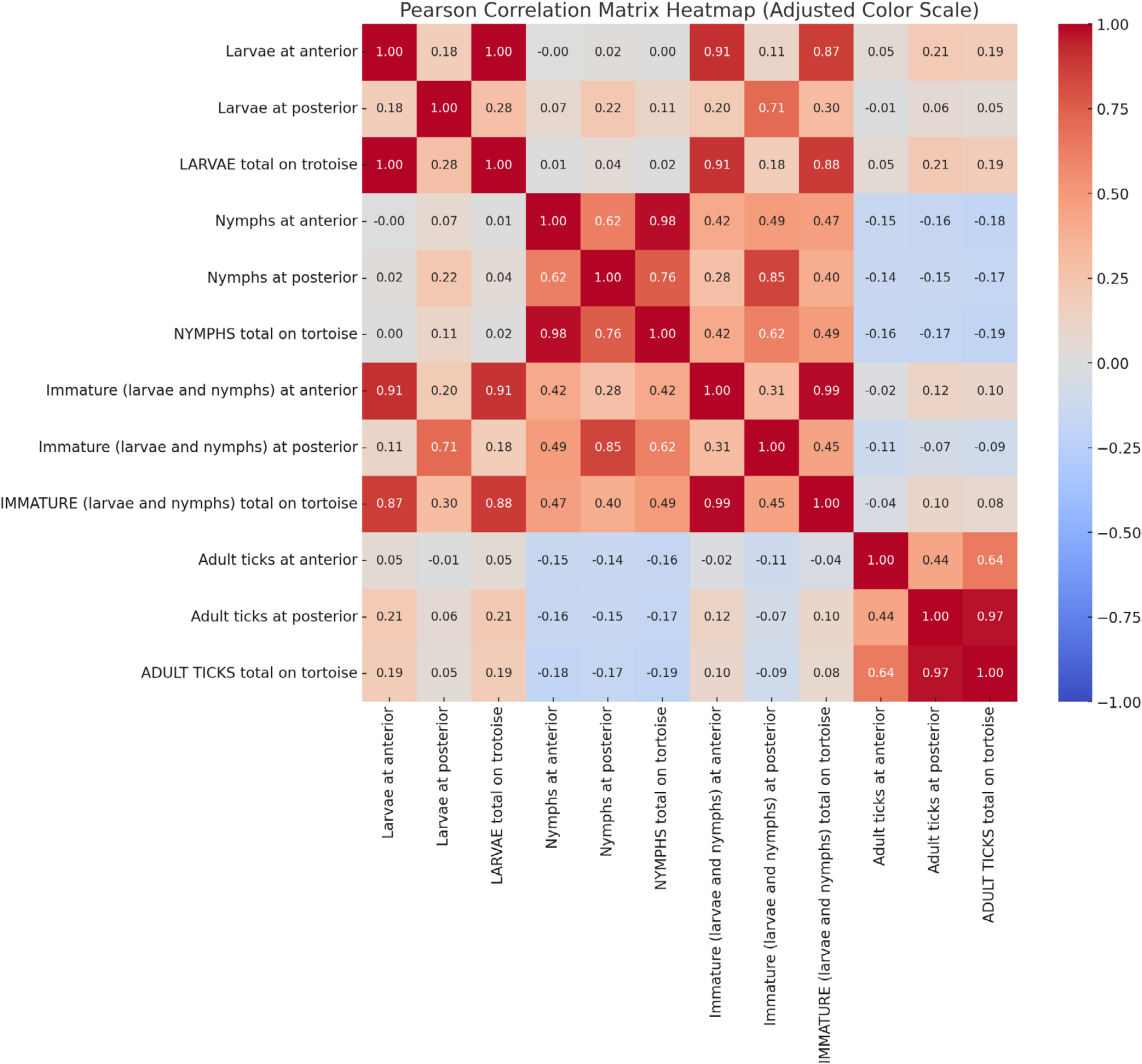
Pearson correlation for attachment site patterns of tick stages on tortoises.

Pearson correlation analysis revealed significant relationships between total tick counts at different developmental stages and their attachment site preferences (Figure 6). Moderate to strong positive correlations were observed between the total number of ticks at a given stage and their attachment preference for the posterior region. A significant correlation was found between total larval counts and larval attachment in the posterior region (*r* = 0.28, *p* < 0.001). Similarly, total nymphal counts strongly correlated with nymphal attachment in the posterior region (*r* = 0.76, *p* < 0.001). These findings support the hypothesis that immature ticks predominantly attach to the anterior areas, while adult ticks exhibit a preference for posterior regions. However, some variations in attachment behaviors indicate that additional factors may influence site selection beyond the developmental stage alone.

### 3.6 Feeding and Mating Phenology in Ticks

No female ticks were recorded in February and March 2021, and only two female ticks were observed on a male tortoise in March 2022. A total of 378 female ticks were counted on tortoises in April 2021 and 2022, but all appeared unfed. The first engorged female ticks were observed in May and continued to be recorded throughout the following months. Similarly, the first mating event was recorded in May, with the ratio of mating females to total females at 9.2%, reaching its annual peak in June (22.2%). Mating was observed in ticks at different feeding stages. Notably, unfed female ticks were observed mating on tortoises, whereas females in different stages of engorgement did not engage in mating on the same animal or vice versa. Pearson correlation analysis revealed a weak negative correlation between larvae counts and the percentage of mating female ticks (*r* ≈ −0.10, *p* ≈ 0.48), and a moderate positive and statistically significant correlation with nymph counts (*r* ≈ 0.40, *p* ≈ 0.002). Male tick counts showed a weak positive, nonsignificant correlation (*r* ≈ 0.12, *p* ≈ 0.36), while female tick counts demonstrated a moderate negative correlation (*r* ≈ −0.24, *p* ≈ 0.071), which approached but did not reach statistical significance (Figure S5).

The observed correlations suggest that developmental stage and sex‐specific factors partially influence tick mating dynamics. The absence of significant associations indicates that inter‐stage relationships alone cannot fully account for the observed patterns. It is plausible that the correlation levels reflect natural phenological cycles and the seasonal overlap among life stages. Therefore, it is likely that additional biotic or abiotic factors, such as environmental cues or host‐related variables, contribute to shaping the mating dynamics of ticks.

## 4 Discussion

This study aimed to analyze the host–parasite relationship between *T. graeca* and *H. aegyptium* , examining the role and limitations of intra‐ and interspecific interactions and morphological and behavioral adaptations. The results demonstrated that infestation levels were higher in adult tortoises than in juveniles, consistent with previous reports (Segura et al. 2019; Najjar et al. 2020). This pattern has been attributed to the fact that adult tortoises forage over longer distances and have a larger surface area, increasing their contact with ticks (Diaz‐Paniagua et al. 1995; Gharbi et al. 2015; Tiar et al. 2016). For adult ticks, a carapace length of 14–20 cm appears optimal, as tortoises within this size range consistently harbored higher tick numbers compared to smaller and larger individuals. Although the underlying mechanisms were not explicitly detailed, previous studies have reported higher infestation rates in sexually mature tortoises than in older individuals (Laghzaoui et al. 2022). Other researchers have explored the ecology of older tortoises (Hailey and Loumbourdis 1990), supporting our nonlinear findings by suggesting that reduced reproductive activity, a possible decline in home range, and changes in skin structure and immunity may contribute to the observed decrease in infestation levels in older hosts.

The results revealed that tortoise sex can be a determinant factor, to varying degrees, in the characterization of tick infestation. Male tortoises carried more ticks than females, consistent with previous studies (Gharbi et al. 2015; Segura et al. 2019; Laghzaoui et al. 2022). However, this sex‐dependent infestation pattern did not consistently trend across all tick developmental stages and time periods. The prevalence, intensity, and abundance of larvae were significantly higher in female tortoises than in males. This finding is not unexpected, as female tortoises exhibit higher philopatry (smaller foraging ranges) and a larger surface area (Diaz‐Paniagua et al. 1995). To fully understand this pattern, the distribution range of larval ticks must be considered. Since female ticks lay eggs in a single clutch, larvae tend to cluster densely in specific questing sites within a given range, which may contribute to the observed differences in infestation patterns.

Since larvae and nymphs that engorge at different times detach from wandering tortoises at random locations within their range, the distribution of questing nymphs and adults of *H. aegyptium* is expected to be more widespread than that of larvae within the habitat. This pattern results in a higher prevalence of nymphs and male ticks on male tortoises, which exhibit larger home ranges and greater roaming activity. However, the monthly infestation patterns of adult ticks exhibited a biphasic character, depending on host sex. A shift in total adult tick intensity was observed toward female tortoises in May and June, whereas infestation levels were higher in male tortoises during the remaining months, and this late‐season difference was primarily associated with male ticks. In fact, by June, the majority of adults present in the habitat have either completed their feeding process and left their hosts (female ticks) or are on the hosts, and new infestation is rare. Based on this phenological pattern, the shift in the number of male ticks on male and female tortoises suggests that adult ticks tend to aggregate on female tortoises during the peak tick mating and feeding period (May and June), which offer a more suitable feeding environment for female ticks due to their larger size and behavioral characteristics. Male ticks follow female ticks (Andrews 1982), and this behavior ultimately drives the aggregation of total adult ticks on female tortoises during this period. However, by mid‐summer, the pattern shifts, increasing total adult tick numbers on male tortoises, as seen in the current study. This transition suggests that female ticks feed intensively and leave the host to lay eggs in the soil during May and June, leaving remaining male ticks to search for new female ticks. As a result, male ticks transfer from female tortoises to males, with larger home ranges and greater mobility, enhancing their mating opportunities with female ticks on different tortoises elsewhere.

The first female ticks of the year were observed in March, with a marked increase in April. However, neither mating nor visible engorgement was recorded until May. The monthly infestation pattern suggested that female infestation duration can exceed 1 month, with considerable inter‐individual variation in this time span. Laboratory trials with *H. aegyptium* (Široký et al. 2011) and field studies on other tortoise‐associated ticks, such as *Amblyomma marmoreum* (Norval 1975) and *Amblyomma sparsum* (Walker and Parsons 1964), support the prolonged infestation period of female ticks. The feeding period of female *H. aegyptium* is longer than that of most other ixodid ticks, including other *Hyalomma* species (Chen et al. 2009), which typically complete a full blood meal within 2 weeks. Most warm‐blooded (endothermic) hosts, such as birds and mammals, are more mobile than tortoises and possess more effective anti‐parasitic defense mechanisms (Bush et al. 2019). Additionally, ticks that parasitize endotherms are constantly exposed to host body temperature, which imposes metabolic stress and likely drives evolutionary pressure for shorter feeding durations. Consequently, it is unsurprising that ticks feeding on endothermic hosts have evolved to feed and detach more quickly. In contrast, *H. aegyptium* on tortoises, which are not exposed to such physiological stressors, may have evolved a more flexible feeding strategy. The absence of thermoregulatory challenges and the relatively stable host environment may have allowed *H. aegyptium* to develop longer feeding durations, optimizing its physiological processes while benefiting from the safer conditions provided by its ectothermic host.

Both unfed, semi‐engorged, and fully engorged *H. aegyptium* females were observed mating. Interestingly, instances where an unfed female engaged in mating while an engorged female did not—or vice versa—were frequently observed on the same tortoises. During the study, six nonmating engorged females were incubated under laboratory conditions, where they successfully laid eggs and the larvae hatched. A similar phenomenon was previously reported in the tortoise tick *Amblyomma sparsum* (Bush et al. 2019). In most ticks, including *Hyalomma* species parasitizing endothermic hosts, females cannot feed to repletion without copulation, as mating is regulated by sex pheromones secreted by females that attract males (Sonenshine and Roe 2014). Typically, one or more males gather around a female, and the mating male tends to remain attached until the female detaches from the host (Sonenshine and Roe 2014; Kar et al. 2021). These findings and the attachment site patterns of females and males on the host suggest the following hypotheses: (i) Different females may secrete sex pheromones at different feeding stages. (ii) Sexual activation may occur at varying times among females coexisting on the same host, potentially influenced by inter‐individual interactions. (iii) Copulation duration may be shorter in *H. aegyptium* than other *Hyalomma* species. (iv) After mating, males may return to their usual aggregation sites, a behavior consistent with that of another tortoise‐parasitizing tick, *Amblyomma humerale* (Labruna et al. 2002).

The protected skin regions of cold‐blooded tortoises provide *H. aegyptium* with a more secure environment than most open‐field conditions. However, this protection does not fully extend to male ticks, which are predominantly attached along the skin‐shell margin in the caudal region of the body. In this position, male ticks face several environmental and mechanical challenges. They are at risk of being compressed between the tortoise shell and skin, experience constant friction from the legs and the hard surface of the hind leg grooves on the shell, and are more vulnerable to external threats, including direct sunlight exposure and desiccation, given that tortoises are nonsweating hosts primarily inhabiting arid environments. This exposure‐related vulnerability suggests that *H. aegyptium* males may have evolved specific morphological adaptations to withstand these conditions. Notably, they exhibit darker, almost black coloration (Estrada‐Peña et al. 2017), which may provide UV protection, a hard, smooth surface with high chitinization, enhancing structural durability, and minimal setae formation, potentially reducing friction‐induced damage. These features appear to be adaptive traits that have evolved to increase survival in the unique attachment orientation required for parasitizing tortoises.

The behavioral, physiological, and morphological adaptations of *H. aegyptium* suggest a highly specialized host adaptation. Without such adaptations, if *H. aegyptium* exhibited feeding behavior similar to other *Hyalomma* species, and if engorged females—often exceeding 1000 mg in weight—fed simultaneously and rapidly on a relatively small host that can harbor large tick infestations, the host could face life‐threatening exsanguination. Supporting this, male ticks, which require minimal blood intake (Sonenshine and Roe 2014), do not occupy the limited feeding space on the small host for extended periods. Instead, they typically remain in the hard‐surfaced marginal areas unsuitable for feeding large females. Considering these factors alongside the segregation pattern of feeding sites (Petney and Al‐Yaman 1985), where the thin‐skinned anterior region is reserved for immature stages, and feeding priority is structured as juveniles, females, and males, it becomes evident that the infestation strategy of *H. aegyptium* exemplifies how natural selection prioritizes reproduction and future generations. Additionally, the prolonged, staggered feeding behavior serves as a strategy to minimize physiological stress on tortoises. However, despite this feeding regulation, there are no constraints on tick population growth, leading to high infestation intensities. Furthermore, the range, morphology, and behavior of both the tick and its tortoise host contribute to this high infestation density. It is a well‐established phenomenon that, in parasite–host relationships that have undergone extensive coevolution, the arms race between species gradually stabilizes, resulting in an equilibrium where parasite‐induced harm to the host is minimized (Dawkins and Krebs 1979; Papkou et al. 2016).

Our results indicate a comprehensive adaptation between *H. aegyptium* and *T. graeca* in terms of behavioral and most morphological characteristics. However, the fact that *H. aegyptium* , a large, highly mobile hunter tick with long, robust legs and high displacement capacity, is specialized to a small tortoise with low mobility and a narrow home range raises questions about their coevolution and specialization depth. This evolutionary paradox is further reinforced by the fact that the size and most morphological characteristics of the *Testudo* genus have remained largely unchanged for 20–30 million years (Vlachos and Rabi 2018). Host and parasite body size are often positively correlated in various parasitic systems, a phenomenon known as Harrison's Rule (Poulin 2007). Although behavioral traits can have a phylogenetic signal (Anderson and Wiens 2017) and exert selective pressure on morphological adaptation (McPeek 1995), morphological adaptations in parasite–host systems are typically acquired much later than behavioral adaptations and tend to have a stronger phylogenetic signal (Blomberg et al. 2003). Consequently, this morphological mismatch suggests that the evolutionary relationship between *H. aegyptium* and *Testudo* may not have originated from a direct, coevolutionary trajectory. However, this inference does not fully explain the numerous species‐specific morphological and complex behavioral adaptations that *H. aegyptium* exhibits toward tortoises. This raises an important question: What host facilitated the evolution of *H. aegyptium* as a large, highly mobile hunter tick before its specialization to tortoises?

The findings of this study, along with current knowledge on the chronological evolutionary history of ticks (e.g., Hoogstraal 1985; Klompen et al. 1996; Sands et al. 2017; Mans et al. 2019), tortoises (e.g., Vlachos and Rabi 2018; Farina et al. 2023), and mammals (e.g., D. A. Apanaskevich 2004; Hu et al. 2005; Kemp 2005), suggest three possible hypotheses regarding the host adaptation of *H. aegyptium* . One possibility is that the behavioral and morphological traits characteristic of the genus *Hyalomma* originally evolved on a now‐extinct giant tortoise that persisted until relatively recent times (20–2 million years ago) (e.g., Pérez‐García et al. 2017). Under this scenario: the species‐level specialization of *Hyalomma* may have diverged into two lineages, with one lineage adapting to *Testudo* ( *H. aegyptium* ) and the other to large mammals (other *Hyalomma* species). The ancestral adaptation to tortoises may have facilitated a gradual transition to *Testudo*, another tortoise group. The size mismatch between *H. aegyptium* and *Testudo* may have been compensated by behavioral adaptations, allowing the tick to persist on a smaller host. The morphological adaptation process may still be incomplete, as the host transition to *Testudo* could be relatively recent in evolutionary terms. Alternatively, morphological adaptation may not occur if behavioral compensation is already sufficient for host exploitation. This hypothesis also explains the ongoing adaptation and evolutionary processes in *Hyalomma* species that parasitize mammals. It further supports the idea that a recent and radical host transition from tortoises to mammals may have occurred, possibly linked to domestication and changes in livestock management (Hoogstraal and Aeschlimann 1982).

A second hypothesis regarding the evolutionary background of *H. aegyptium* suggests that the ancestral *Hyalomma* species may have evolved on medium‐ to large‐sized tortoise ancestors, which first appeared between 252 and 66 million years ago (Mya) (Farina et al. 2023). Under this scenario, *H. aegyptium* , having already completed its size‐related and morphological evolution, may have radiated from these large tortoises to the *Testudo* ancestors, which underwent a miniaturization process approximately 30–20 Mya and persisted with minimal morphological changes to the present day (Vlachos and Rabi 2018). While this hypothesis accounts for certain aspects of *H. aegyptium* 's evolutionary trajectory, it does not fully explain the phylogenetic immaturity of other *Hyalomma* species. Additionally, there are discrepancies regarding the suggested timeline for *Hyalomma* evolution: the origin of the genus is estimated to date back to 50–63 Mya (De la Fuente 2003; Mans et al. 2019). The divergence of *Hyalomma* members is thought to have occurred around 36 Mya. *Hyalomma aegyptium* is hypothesized to have diverged from other members approximately 27 Mya (Sands et al. 2017).

A third hypothesis speculates that the ancestor of *Hyalomma* may have initially evolved in mammals, with *H. aegyptium* later transitioning from a large mammal to a tortoise. However, this scenario presents a temporal mismatch: Stem mammals, which had morphological and physiological characteristics quite different from those of today's mammals, are estimated to have first appeared at around 165 (150–250) Mya (Crompton and Jenkins 1973; Hu et al. 2005; Rougier et al. 2007). Significant radiation of this relatively small common ancestor (Baker et al. 2015) is believed to have occurred around 35 Mya (Kemp 2005). For most of their evolutionary history, mammals remained small‐sized (typically mouse‐sized, rarely exceeding the size of a cat), with medium‐ to large‐bodied mammals emerging only 2–3 Mya (Kemp 2005). Additionally, a mammal‐to‐tortoise transition would represent an unusual and highly complex evolutionary shift, as it would require a radical and seemingly retrogressive adaptation, particularly given the size incompatibility between large‐bodied mammals and relatively small tortoises.

## 5 Conclusion

This study's findings and evolutionary analyses suggest that the behavioral, morphological, and physiological characteristics of *Hyalomma aegyptium* reflect a finely tuned adaptation to *Testudo graeca* . Some incompatibilities, such as size discrepancies and high reproductive capacity, which potentially pose a significant threat to the host, appear to be compensated by sophisticated behavioral adjustments. These adaptations, in turn, indicate a contribution to the establishment of a well‐established parasite–host relationship that appears to have reached a stable and mutual equilibrium. However, although the comprehensive adaptive traits suggest a deep‐rooted coevolutionary background and even cospeciation between the tick and tortoises, some morphological incompatibility in terms of parasite–host adaptation more strongly supports the hypothesis that the primary speciation process of *H. aegyptium* might be completed in a giant tortoise in history, rather than within the genus *Testudo*. We believe that presenting these evolutionary suggestions with specified genomic analyses will provide critical data for understanding evolution in both ticks and many other living systems.

## Author Contributions

**Sirri Kar:** conceptualization (lead), investigation (lead), methodology (lead), resources (lead), supervision (lead), writing – original draft (lead). **Baris Donmez:** investigation (supporting), visualization (supporting). **Bugrahan Regaip Kilinc:** formal analysis (lead), visualization (lead). **Zafer Sakaci:** investigation (equal), writing – review and editing (supporting). **Sengul Talay:** investigation (supporting), visualization (supporting). **Dennis Bente:** formal analysis (supporting), methodology (supporting), writing – review and editing (supporting). **Agustin Estrada‐Peña:** formal analysis (supporting), methodology (supporting), validation (supporting), writing – review and editing (equal).

## Ethics Statement

This study was carried out within the framework of legal permissions received from the Ministry of Agriculture, Türkiye (E‐97662369‐325.04.02‐2517603).

## Consent

The authors have nothing to report.

## Conflicts of Interest

The authors declare no conflicts of interest.

## Supporting information


**Figure S1:** Monthly proportions of male and female tortoises.
**Figure S2:** Violin plot of monthly tortoise size distributions.
**Figure S3:** Tick counts by carapace length of tortoise, stratified by tick stage.
**Figure S4:** Fully engorged *Hyalomma aegyptium* female (a), and engorging females on the skin and males at the caudal axillar depression on tortoise (b).
**Figure S5:** Pearson correlation for evaluating the relationship between tick developmental stages and number of mating females.

**Table S1:** Monthly infestation characteristics of the *Hyalomma aegyptium* larvae and nymphs in tortoises.
**Table S2:** Monthly infestation characteristics of female and male *Hyalomma aegyptium* in tortoises.
**Table S3:** Monthly characteristics of *Hyalomma aegyptium* infestation in the male tortoises.
**Table S4:** Monthly characteristics of *Hyalomma aegyptium* infestation in the female tortoises.

## Data Availability

Data supporting the conclusions of this article are included within the article and its additional files.
