# Supplementary material for: Behavioral and Morphological Adaptations of Tortoise Tick Hyalomma aegyptium to Testudo graeca: Evidence for Complex Evolutionary History
Source: Ecol Evol. 2025 Aug 14;15(8):e71995. doi: 10.1002/ece3.71995 (PMC12351805; doi:10.1002/ece3.71995)
Supplement: Supplementary file 1 — Figure S1: Monthly proportions of male and female tortoises. Figure S2: Violin plot of monthly tortoise size distributions. Figure S3: Tick counts by carapace length of tortoise, stratified by tick stage. Figure S4: Fully engorged Hyalomma aegyptium female (a), and engorging females on the skin and males at the caudal axillar depression on tortoise (b). Figure S5: Pearson correlation for evaluating the relationship between tick developmental stages and number of mating females. [file ECE3-15-e71995-s002.pdf]

Ecology and Evolution

Behavioral and Morphological Adaptations of Tortoise tick *Hyalomma aegyptium* to *Testudo graeca*: Evidence for Complex Evolutionary History

Sirri Kar, Baris Donmez, Bugrahan Regaip Kilinc, Zafer Sakaci, Sengul Talay, Dennis Bente, Agustin Estrada-Pena

Supplementary Figures

Suppl. Figure 1. Monthly proportions of male and female tortoises.

Suppl. Figure 2. Violin plot of monthly tortoise size distributions.

Suppl. Figure 3 Tick counts by carapace length of tortoise, stratified by tick stage.

Suppl. Figure 4 Fully engorged *Hyalomma aegyptium* female (a), and engorging females on the skin and males at the caudal axillar depression on tortoise (b).

Suppl. Figure 5 Pearson correlation for evaluating the relationship between tick developmental stages and number of mating females.

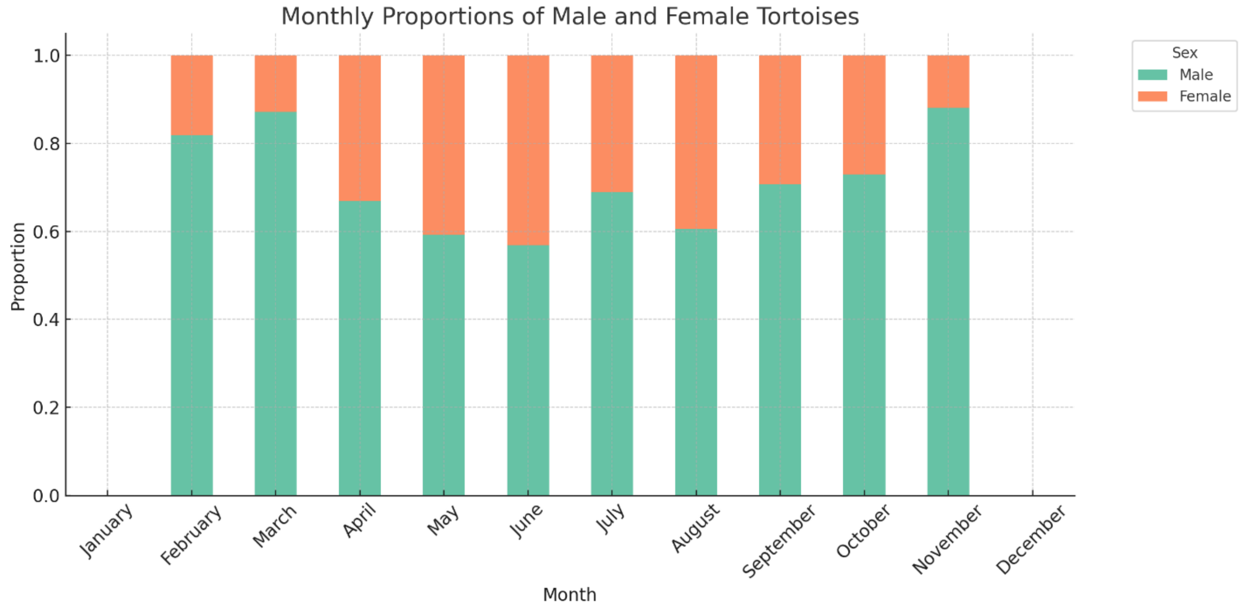

Suppl. Figure 1. Monthly proportions of male and female tortoises.

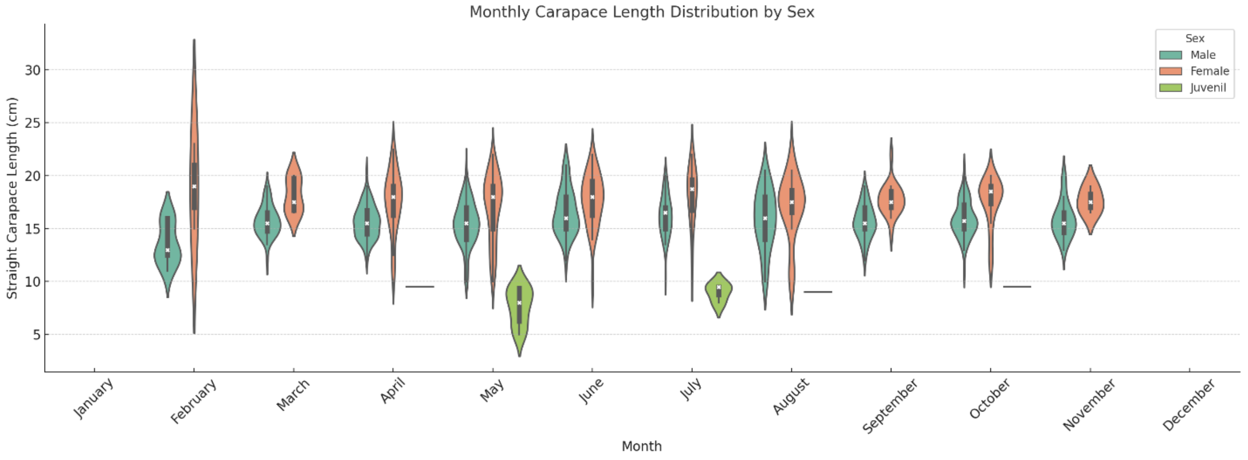

Suppl. Figure 2. Violin plot of monthly tortoise size distributions.

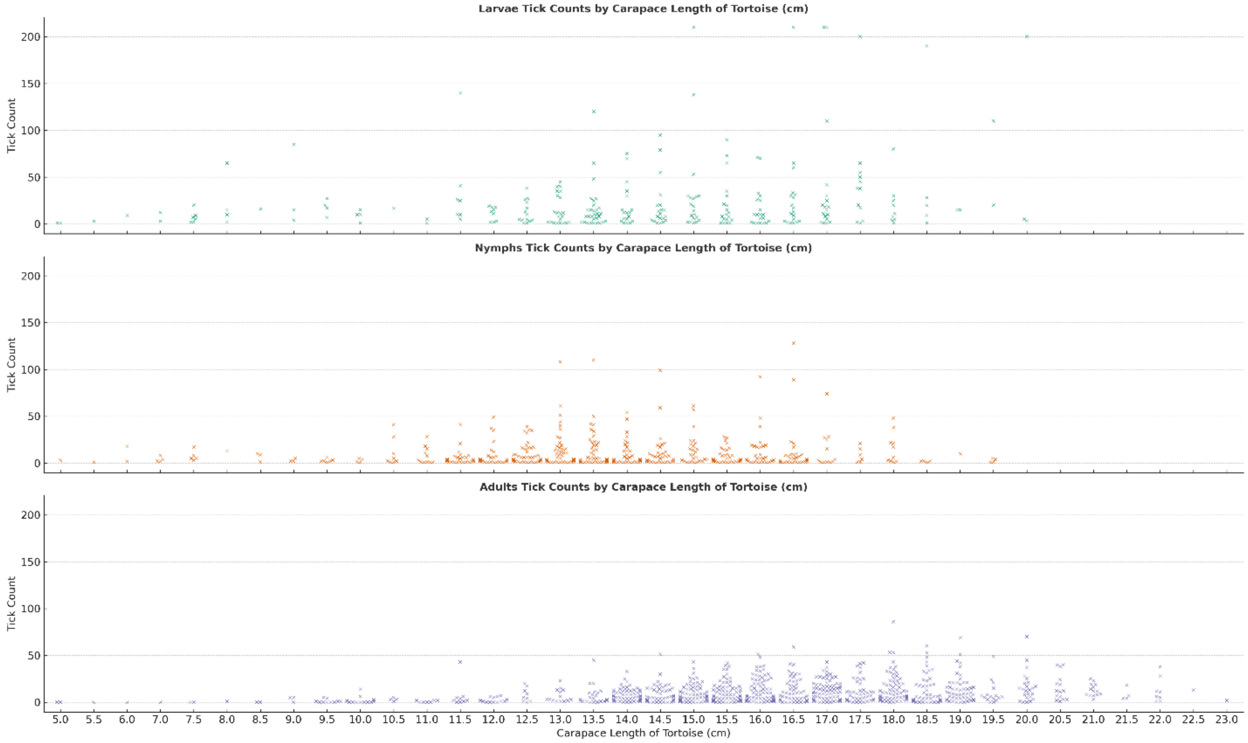

Suppl. Figure 3 Tick counts by carapace length of tortoise, stratified by tick stage.

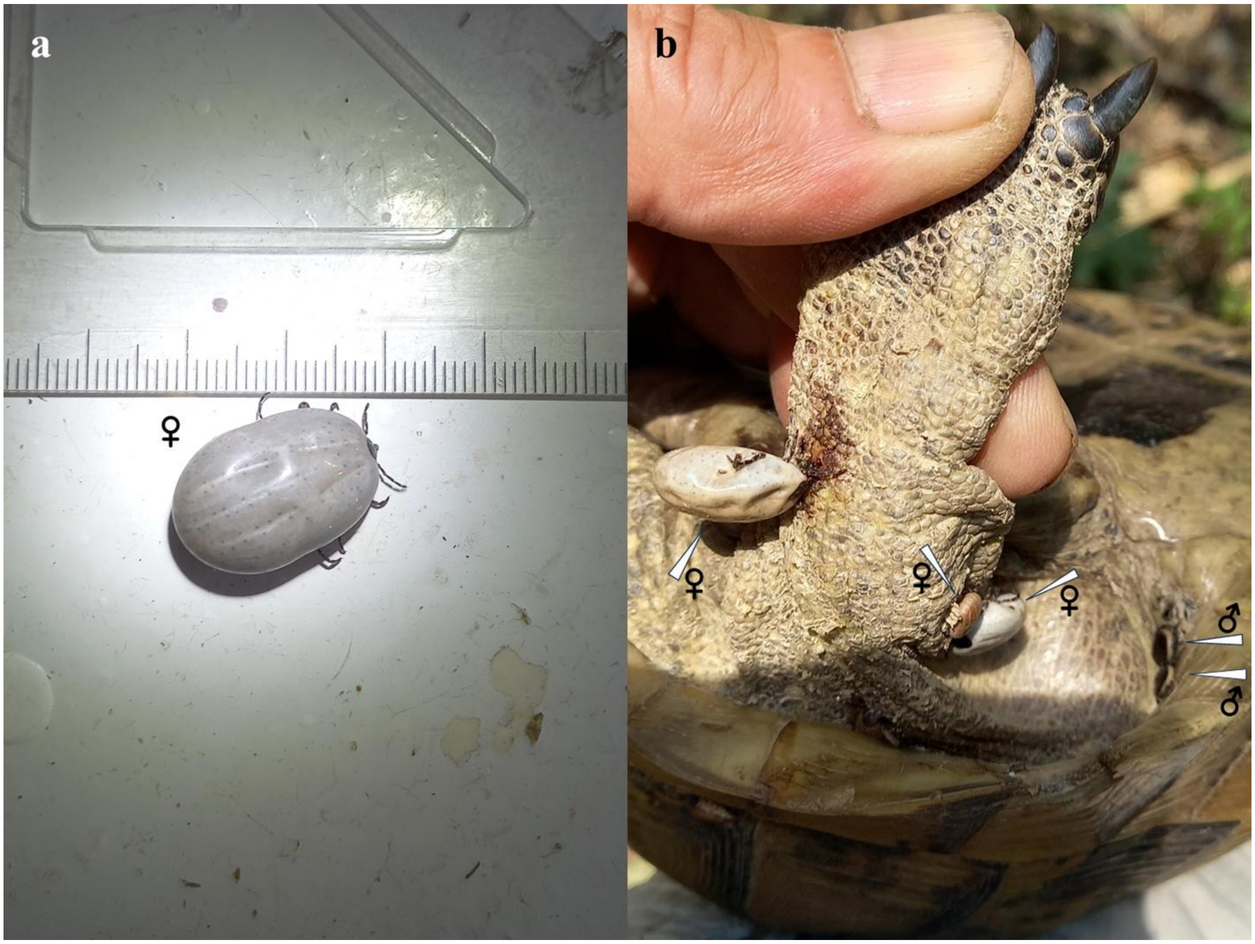

Suppl. Figure 4 Fully engorged *Hyalomma aegyptium* female (a), and engorging females on the skin and males at the caudal axillar depression on tortoise (b).

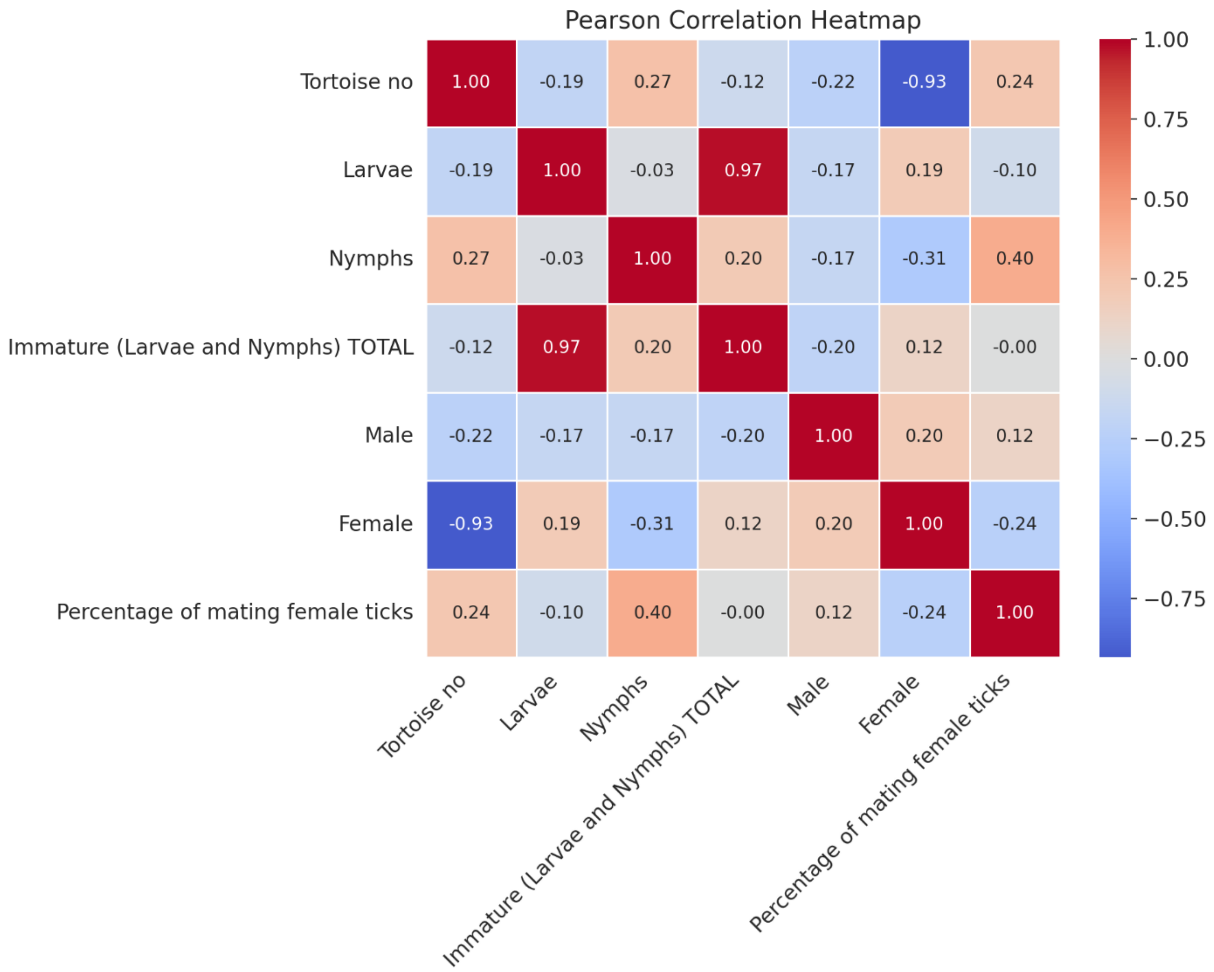

Suppl. Figure 5 Pearson correlation for evaluating the relationship between tick developmental stages and number of mating females.
