# Supplementary material for: Behavioral and Morphological Adaptations of Tortoise Tick Hyalomma aegyptium to Testudo graeca: Evidence for Complex Evolutionary History
Source: Ecol Evol. 2025 Aug 14;15(8):e71995. doi: 10.1002/ece3.71995 (PMC12351805; doi:10.1002/ece3.71995)
Supplement: Supplementary file 2 — Table S1: Monthly infestation characteristics of the Hyalomma aegyptium larvae and nymphs in tortoises. Table S2: Monthly infestation characteristics of female and male Hyalomma aegyptium in tortoises. Table S3: Monthly characteristics of Hyalomma aegyptium infestation in the male tortoises. Table S4: Monthly characteristics of Hyalomma aegyptium infestation in the female tortoises. [file ECE3-15-e71995-s001.pdf]

Ecology and Evolution

Behavioral and Morphological Adaptations of Tortoise tick *Hyalomma aegyptium* to *Testudo graeca*: Evidence for Complex Evolutionary History

Sirri Kar, Baris Donmez, Bugrahan Regaip Kilinc, Zafer Sakaci, Sengul Talay, Dennis Bente, Agustin Estrada-Pena

Supplementary Tables

Suppl. Table 1 Monthly infestation characteristics of the *Hyalomma aegyptium* larvae and nymphs in tortoises.

Suppl. Table 2 Monthly infestation characteristics of female and male *Hyalomma aegyptium* in tortoises.

Suppl. Table 3 Monthly characteristics of *Hyalomma aegyptium* infestation in the male tortoises.

Suppl. Table 4 Monthly characteristics of *Hyalomma aegyptium* infestation in the female tortoises.

Suppl. Table 1 Monthly infestation characteristics of the *Hyalomma aegyptium* larvae and nymphs in tortoises.

Month	No. tortoises	Larvae		Nymphs	
		No. infested tortoises (Prevalence)	Mean intensity ±SD (range)	No. infested tortoises (Prevalence)	Mean intensity ±SD (range)
Jan	Tortoise not found				
Feb	11	-	-	4 (36.4)	2.5±1.5 (1-4)
Mar	54	-	-	4 (7.1)	1.5±1.0 (1-3)
Apr	156	1 (0.6)	1	52 (33.3)	2.5±2.1 (1-11)
May	247	85 (34.4)	19.9±28.0 (1-190)	89 (36.0)	2.1±1.6 (1-9)
June	81	35 (43.2)	33.7±49.8 (1-210)	26 (32.1)	1.9±1.2 (1-6)
July	80	61 (76.3)	41.8±53.0 (1-210)	61 (76.3)	7.2±14.5 (1-110)
Aug	73	40 (54.8)	19.0±18.5 (1-90)	69 (94.5)	20.8±19.7 (1-108)
Sept	75	42 (56.0)	7.3±9.8 (1-53)	74 (98.7)	24.1±21.8 (1-128)
Oct	76	14 (18.4)	2.0±2.8 (1-12)	72 (94.7)	10.3±12.5 (1-92)
Nov	25	2 (8.0)	4.0±2.0 (2-6)	23 (92.0)	7.0±6.7 (1-28)
Dec	Tortoise not found				
**Total**	878	280 (31.9)	23.3±37.2 (1-210)	474 (54.0)	10.4±16.0 (1-128)

Suppl. Table 2 Monthly infestation characteristics of female and male *Hyalomma aegyptium* in tortoises.

Month	No. tortoises	Male		Female	
		**No. infested tortoises (Prevalence-%)**	**Mean intensity (range)**	**No. infested tortoises (Prevalence-%)**	**Mean intensity (range)**
Jan	Tortoise not found				
Feb	11	9 (81.8)	4.4±4.3 (1-14)	-	-
Mar	54	42 (77.8)	3.6±3.0 (1-13)	1 (1.9)	2.0
Apr	156	146 (93.6)	11.1±7.8 (1-41)	106 (67.9)	3.6±2.8 (1-14)
May	247	219 (88.7)	14.3±10.2 (1-70)	207 (83.8)	6.7±5.4 (1-30)
June	81	81 (100)	10.9±8.0 (1-35)	57 (70.4)	5.3±5.5 (1-28)
July	80	77 (96.3)	7.2±5.6 (1-27)	50 (62.5)	4.1±4.3 (1-25)
Aug	73	57 (78.1)	3.7±3.4 (1-16)	47 (64.4)	2.7±2.6 (1-14)
Sept	75	51 (68.0)	3.2±3.2 (1-20)	12 (16.0)	1.6±0.9 (1-3)
Oct	76	61 (80.3)	3.1±3.4 (1-24)	5(6.6)	1.0±0.0 (1-1)
Nov	25	24 (96.0)	4.0±2.9 (1-11)	5 (20.0)	1.2±0.4 (1-2)
Dec	Tortoise not found				
**Total**	878	767 (87.4)	9.2±8.6 (1-70)	490 (55.8)	5.0±4.8 (1-30)

Suppl. Table 3 Monthly characteristics of *Hyalomma aegyptium* infestation in the male tortoises.

Month	n	Male tortoise				
		Ticks (prevalence/mean intensity ±SD)				
		Larvae	Nymphs	Males	Females	Total adults
Jan	Tortoise not found					
Feb	9	-	44.4/2.5±1.5	77.8/5.0±4.8	-	77.8/5.0±4.8
Mar	47	-	8.5/1.5±1.0	78.7/3.7±3.2	4.3/2	78.7/3.9±3.1
Apr	103	1.0/1.0	37.9/2.5±2.1	98.1/12.5±8.4	71.8/3.7±2.6	98.1/16.2±11.2
May	138	31.2/17.3±18.4	37.7/1.9±1.3	95.7/16.3±10.9	91.3/6.6±4.9	97.1/28.5±19.2
June	46	41.3/28.5±29.9	28.3/1.8±0.6	100/9.4±5.9	58.7/4.4±4.2	100/11.9±8.7
July	53	71.7/34.6±43.2	75.5/5.3±5.8	98.1/6.7±4.9	64.2/4.2±4.8	98.1/9.4±8.3
Aug	43	51.2/15.7±13.3	95.4/22.0±20.5	76.7/3.9±3.2	60.5/2.6±3.1	90.7/5.1±4.4
Sept	53	64.2/7.9±10.7	100/29.2±23.3	67.9/3.9±3.6	20.8/1.6±0.9	73.6/4.1±3.7
Oct	54	20.4/2.2±3.1	94.4/11.2±8.3	87.0/3.3±3.7	7.4/1.0	87.0/3.4±3.7
Nov	22	9.1/4.0±2.0	90.9/6.9±6.7	95.5/4.2±3.1	22.7/1.2±0.4	100/4.3±3.1
Dec	Tortoise not found					
**Total**	568	29.9/19.1±27.6 (range: 1-210)	55.8/11.4±16.4 (range: 1-128)	90.1/9.5±9.0 (range: 1-70)	54.2/4.7±4.4 (range: 1-27)	91.9/12.2±12.1 (range: 1-86)

Suppl. Table 4 Monthly characteristics of *Hyalomma aegyptium* infestation in the female tortoises.

Month	n	Female tortoise				
		Ticks (prevalence/mean intensity ±SD)				
		Larvae	Nymphs	Males	Females	Total adults
Jan	Tortoise not found					
Feb	2	-	-	100/2.5±0.5	-	100/2.5±0.5
Mar	7	-	-	71.4/2.6±1.5	-	71.4/2.6±1.5
Apr	51	-	23.5/2.6±2.5	86.3/8.0±4.9	62.7/3.2±3.1	90.2/10.5±8.1
May	95	33.7/27.9±39.0	37.9/2.4±2.0	89.5/11.5±7.9	85.3/7.0±6.1	95.8/27.7±31.5
June	35	45.7/39.9±65.5	37.1/1.9±1.6	100/12.8±9.8	85.7/6.1±6.3	100/18.1±14.7
July	24	87.5/57.6±65.9	75.0/12.1±24.5	95.8/8.9±6.7	62.5/3.9±3.0	11.4±9.0
Aug	28	64.3/23.1±22.7	92.9/19.8±18.6	85.7/3.5±3.6	67.9/2.5±1.7	96.4/4.8±3.9
Sept	22	36.4/5.0±3.3	95.5/11.3±8.4	68.2/1.5±0.6	4.6/1	68.2/1.6±0.7
Oct	20	15.0/1.3±0.5	100/8.1±19.4	65.0/2.6±2.0	5.0/1	65.0/2.7±2.0
Nov	3	-	100/8.0±6.5	100/2.7±1.3	-	100/2.7±1.3
Dec	Tortoise not found					
**Total**	287	34.2/32.6±49.7 (range: 1-210)	51.9/8.7±15.4 (range: 1-110)	86.8/8.6±7.7 (range: 1-39)	62.4/5.3±5.4 (range: 1-30)	89.2/12.1±11.7 (range: 1-69)
